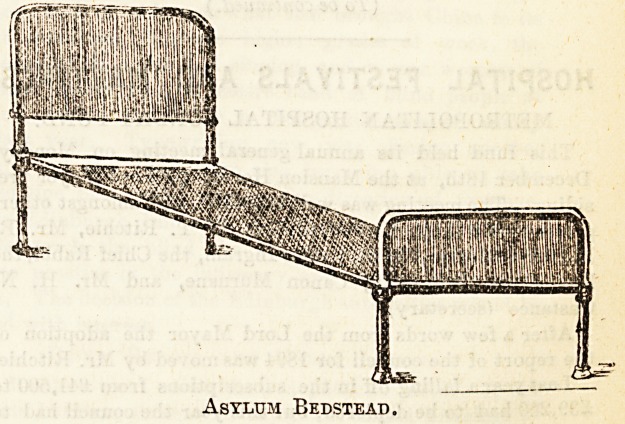# Bedsteads and Bedding

**Published:** 1894-12-29

**Authors:** 


### PRACTICAL DEPARTMENTS.

### BEDSTEADS AND BEDDING—(*Continued*).

Bedsteads for hospital and sick-room use should never be wider than absolutely necessary for the comfort of its occupant. Three to three and a half feet is the best width. Miss

Bedstead with Back Rest.

Lückes, in her "Lectures on Nursing," recommends that where there is "an idea of affording the patient relief by changing him from side to side, this plan can be executed infinitely better by means of two smaller beds that can be put next each other for the purpose of lifting the patient to a fresh place, and the bed which he has just vacated can then be moved away without disturbing him," the nurse remaining able to get at her patient with far greater comfort to

Asylum Bedstead.

both than if the manœuvre were to be carried out on a large bed.

For length six and a half feet is generally the best for an adult, and it is essential for comfort that there should be a foot-rail of some kind. The slipping sensation engendered by its absence is very uncomfortable for a sick person, and its presence prevents the possibility of any displacement of the mattress.

Many plans have been attempted to combine a comfortable back-rest with the bedstead itself, and some are distinctly good. The first illustration we give shows a bedstead made by Messrs. Heal and Son, of Tottenham Court Road, whose name is sufficient guarantee for its practical excellence. The "rest" portion of the frame is raised by the handle at the foot, and the elevating can be done with perfect ease and without any objectionable jerking. This bedstead is intended for hospital use, and is very reasonable in price, while thoroughly strong and well made.

We noted a new idea in this direction in The Hospital for August 4th, 1894, in the "Cambridge Bedstead," itself the invention of an invalid, the Rev. W. Goodliffe. Here the back rest is achieved by the head rail being made moveable, adjusted easily to the desired angle, and secured in its place by a nut. This has the advantage that the occupant of the bed can manage the change of position for himself, with little exertion.

It is not so very long since the days when flock beds were the rule in hospital wards, and if they possessed at least the merit of cleanliness, being easily washed and replenished, for lumpiness and discomfort they were hard to beat. The gradual and now almost universal substitution of galvanised wire springs and hair mattresses is a matter for congratulation, and these leave nothing to be desired on sanitary grounds, the springs being very easily kept clean. Messrs. Heal have much to show in the way of mattresses of various kinds, and those sold by this firm are thoroughly to be relied on, seeing that they supply none which have not been made on their own premises, and are guaranteed.

For some bedsteads tightly stretched sacking is used instead of the galvanised wire frame, and this has to recommend it the fact that it can be so quickly washed and replaced.

For special use in hot climates rods and poles may be had for fixing to the ordinary bedstead to support mosquito curtains, and these same rods and poles do excellently for the steam tents which are so frequently needed in medical wards.

Our second illustration shows a "Chorlton's" spring bedstead intended for asylum use. It is made in three parts; with head and foot pieces of sheet iron. There are no unnecessary corners whereby the patient can inflict damage upon himself, and the "stump" feet are of wood for the sake of extra steadiness. This bed is made by Messrs. Shoolbred and Co., Tottenham Court Road, by whose permission we give the accompanying sketch.

(*To be continued.*)

## Figures and Tables

**Figure f1:**
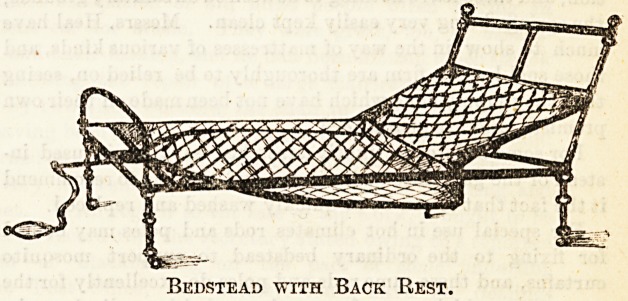


**Figure f2:**